# Drug Development Against the Major Diarrhea-Causing Parasites of the Small Intestine, *Cryptosporidium* and *Giardia*

**DOI:** 10.3389/fmicb.2015.01208

**Published:** 2015-11-19

**Authors:** Yukiko Miyamoto, Lars Eckmann

**Affiliations:** Department of Medicine, University of California at San Diego, La JollaCA, USA

**Keywords:** diarrhea, neglected diseases, protozoan parasites, cryptosporidiosis, giardiasis, antimicrobial drugs, drug development

## Abstract

Diarrheal diseases are among the leading causes of morbidity and mortality in the world, particularly among young children. A limited number of infectious agents account for most of these illnesses, raising the hope that advances in the treatment and prevention of these infections can have global health impact. The two most important parasitic causes of diarrheal disease are *Cryptosporidium* and *Giardia*. Both parasites infect predominantly the small intestine and colonize the lumen and epithelial surface, but do not invade deeper mucosal layers. This review discusses the therapeutic challenges, current treatment options, and drug development efforts against cryptosporidiosis and giardiasis. The goals of drug development against *Cryptosporidium* and *Giardia* are different. For *Cryptosporidium*, only one moderately effective drug (nitazoxanide) is available, so novel classes of more effective drugs are a high priority. Furthermore, new genetic technology to identify potential drug targets and better assays for functional evaluation of these targets throughout the parasite life cycle are needed for advancing anticryptosporidial drug design. By comparison, for *Giardia*, several classes of drugs with good efficacy exist, but dosing regimens are suboptimal and emerging resistance begins to threaten clinical utility. Consequently, improvements in potency and dosing, and the ability to overcome existing and prevent new forms of drug resistance are priorities in antigiardial drug development. Current work on new drugs against both infections has revealed promising strategies and new drug leads. However, the primary challenge for further drug development is the underlying economics, as both parasitic infections are considered Neglected Diseases with low funding priority and limited commercial interest. If a new urgency in medical progress against these infections can be raised at national funding agencies or philanthropic organizations, meaningful and timely progress is possible in treating and possibly preventing cryptosporidiosis and giardiasis.

The two most frequent parasitic causes of diarrheal disease worldwide are *Cryptosporidium* and *Giardia*. Other parasites, such as *Entamoeba histolytica*, which is highly invasive and causes amebic colitis (as discussed in other chapters of this volume), and the coccidia, *Cystoisospora belli*, and *Cyclospora cayetanensis*, have clinical relevance but are less common causes of diarrheal illness. For instance, the two coccidian parasites are mostly associated with chronic diarrhea in HIV-positive individuals (Dash et al., 2013; Mathur et al., 2013) and other forms of immunodeficiency, but are less important causes of diarrhea in immunocompetent individuals. This review focuses on the therapeutic challenges, current treatment options, and ongoing drug development efforts against cryptosporidiosis and giardiasis.

## Cryptosporidium

*Cryptosporidium* is a major cause of diarrheal disease in humans and animals. It became clinically first known as a causative agent of profuse and persistent diarrhea and morbidity in immunocompromised patients, particularly those with acquired immunodeficiency syndrome, and as a major cause of waterborne diarrheal disease in outbreaks in otherwise healthy individuals. More recently, large population studies have shown that *Cryptosporidium* is among the five leading causes of diarrheal disease in young children worldwide (Checkley et al., 2015), underlining the urgency of addressing the medical needs posed by this parasite, particularly since current treatment options are severely limited.

### Parasite

*Cryptosporidium* belongs to the phylum of apicomplexan protists, along with *Plasmodium* and *Toxoplasma*, although it displays significant differences to those parasites. Most notably, it has lost the phylum-defining apicomplexan plastid and lacks mitochondria. Genome comparisons suggest that *Cryptosporidium* is more closely related to gregarines, intestinal protozoa of invertebrates (Carreno et al., 1999; Ryan and Hijjawi, 2015). The parasite is an obligate endosymbiont, depending on invasion of host cells for numerous metabolic functions. Consistent with the exploitation of this metabolically rich biological niche, it has a relatively small eukaryotic genome of ∼9 Mb with ∼4,000 genes (Abrahamsen et al., 2004; Xu et al., 2004). *Cryptosporidium* represents a species complex comprising at least 27 individual species and over 40 genotypes with varying degrees of host specificity (Ryan and Hijjawi, 2015). Humans can be infected by nearly 20 of these species, but only two, *Cryptosporidium hominis* and *C. parvum*, cause the majority of clinically relevant infections. *C. hominis* is limited to humans, so the infectious cycle is strictly anthroponotic, while *C. parvum* has several subtypes of which some are human-specific and others have a broader host range and zoonotic transmission. Importantly, new drugs must be active against *C. parvum* and *C. hominis* as both species have worldwide distribution.

**FIGURE 1:**
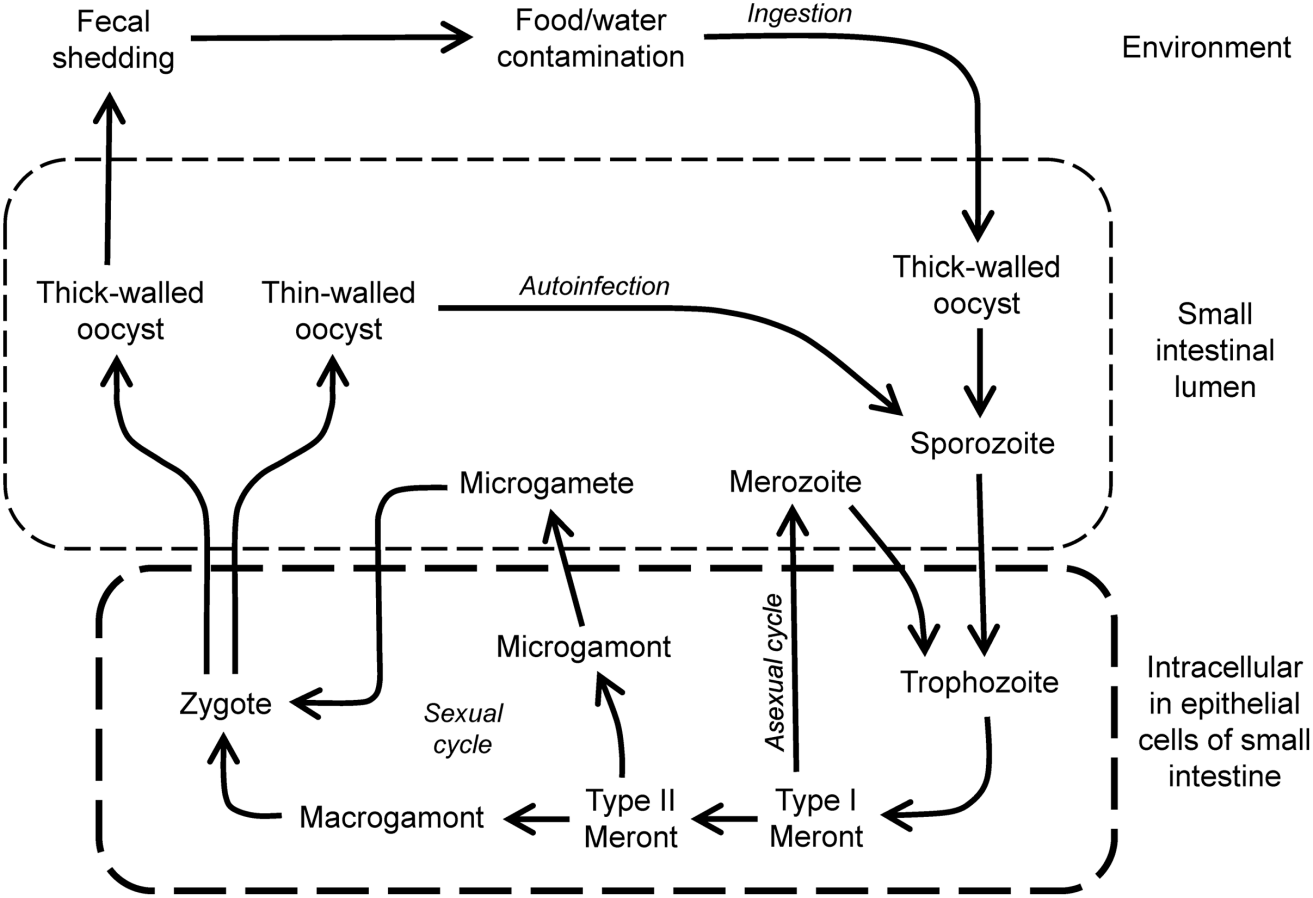
**Life cycle of *Cryptosporidium*.** Infection is initiated by ingestion of thick-walled and environmentally resistant oocysts that give rise to sporozoites in the lumen of the small intestine. These invade epithelial cells to form trophozoites in the apical domain of the cells. Growth occurs by asexual multiplication, leading to further cycles of infection and growth, or sexual multiplication involving gamonts and gametes, and leading to fertilized zygotes. These can differentiate into thin-walled oocysts that can initiate further rounds of autoinfection, or to thick-walled oocysts that are shed in the feces.

The entire *Cryptosporidium* life cycle occurs in a single host (monoxenous) and involves both asexual multiplication and sexual reproduction (Laurent et al., 1999) (**Figure 1**). Infectious oocysts are ingested by the host, and sporozoites emerge from the oocysts upon exposure to acidic conditions followed by neutralization and exposure to pancreatic enzymes and bile (Smith et al., 2005). Sporozoites attach to intestinal epithelial cells, are enveloped by the host cell apical cell membrane, and differentiate into spherical trophozoites, which occupy a location that is commonly described as intracellular but extracytoplasmic (Smith et al., 2005). The parasites reside in a parasitophorous vacuole, which contains membrane components from the host and parasite, and allows acquisition of nutrients from the host cell (Tzipori and Griffiths, 1998). Importantly, the parasite is completely covered by host cell membrane during its epithelial growth phase, so drugs have to cross this membrane to be effective at that stage of the growth cycle.

During maturation of the *Cryptosporidium* trophozoite, asexual multiplication occurs and results in the formation of a type I schizont that contains six to eight merozoites. Rupture of the schizont results in the release of merozoites that, in turn, can invade adjacent host epithelial cells, where they develop subsequently into type I schizonts, leading to further rounds of asexual multiplication, or into type II schizonts, which initiate sexual reproduction by differentiating into male microgamonts or female macrogamonts (Current and Reese, 1986). Male microgamonts release microgametes that can fertilize the macrogametes inside the female macrogamont. After fertilization, two types of oocysts form, thin-walled oocysts, which are important in reinfection of the host and expansion of parasite numbers, and thick-walled oocysts, which exit the intestinal tract and are infectious for new hosts.

### Pathogenesis and Disease

Transmission occurs by the fecal–oral spread of oocysts. In particular, fecal contamination of drinking water can serve as a vehicle for transmission of oocysts and is a substantial public health concern. Large-scale outbreaks have been associated with contamination of community drinking water (Widerstrom et al., 2014; Painter et al., 2015). *Cryptosporidium* invades and resides for major parts of its life cycles within epithelial cells, most commonly in the small intestine. The parasite can be viewed as a “minimally invasive” mucosal pathogen, because it does not usually penetrate into the deeper mucosal layers. This restricted epithelial localization has potential implications for drug design, as it raises the possibility that orally administered drugs might be effective locally in the intestine without extensive systemic absorption. Under conditions of immunodeficiency, *Cryptosporidium* infection can be more widespread and involve epithelial cells of the biliary tract, pancreatic duct, stomach, esophagus, and even respiratory tract (Chen et al., 2002). Under these conditions, systemic drug absorption is probably required, as a drug with limited intestinal activity would be unlikely to reach these other mucosal sites (Abubakar et al., 2007).

Clinical manifestations of *Cryptosporidium* infection, which occur after an incubation period of 2–14 days, include watery and often profuse diarrhea, as well as abdominal cramps, nausea, vomiting, weight loss, and a low-grade fever (Chen et al., 2002). In immunocompetent individuals, disease is usually self-limited lasting 1–3 weeks, whereas the illness is typically prolonged in immunocompromised hosts. In addition, in severe infections, malabsorption can be present due to decreased absorptive surface, which can contribute to the wasting syndrome in infected AIDS patients (Hunter and Nichols, 2002). Immunocompromised patients with bile duct infection may also present with jaundice secondary to biliary tract obstruction or with symptoms of pancreatitis. Furthermore, in young children, particularly if malnourished, the infectious diarrhea can be extensive and associated with significant mortality (Hunter and Nichols, 2002). In fact, cryptosporidiosis has recently been identified as the second leading cause (after rotavirus) of diarrheal disease in children <5 years in developing countries (Kotloff et al., 2013; Liu et al., 2014). With increasing use of the highly effective rotavirus vaccine it can be expected that *Cryptosporidium* will soon become the most important worldwide cause of childhood diarrhea.

Several mechanisms are thought to contribute to the pathogenesis of *Cryptosporidium*-induced diarrhea. In patients having large volume diarrhea, a secretory process appears to be important. An enterotoxic activity released by the parasite has been described (Guarino et al., 1995), but these findings remain controversial. In animal models of *C. parvum* infection, glucose-stimulated sodium absorption was inhibited and this paralleled the extent of villous and epithelial cell damage (Kapel et al., 1997). In addition, increased mucosal prostaglandin production (e.g., PGE_2_ and PGI_2_), which can inhibit neutral NaCl absorption and result in secretory diarrhea, has been demonstrated in the porcine model (Argenzio et al., 1990). Such effects may be due, in part, to PGI_2_ acting on components of the enteric nervous system (i.e., cholinergic and VIPergic neuronal pathways), and a direct action of PGE_2_ on enterocytes (Argenzio et al., 1993, 1996). The mechanisms that lead to increased prostaglandin production and the cellular source of the prostaglandins are not known, although the latter may include mesenchymal and/or epithelial cells (Laurent et al., 1998). In addition, resident and recruited leukocytes in the mucosa (e.g., macrophages) have the potential to produce high levels of prostaglandins, and may also be a source of prostaglandin production during *C. parvum* infection. Alterations in intestinal permeability may also play a role in the diarrhea in *C. parvum*-infected individuals (Zhang et al., 2000).

### Current Treatment Options

Current therapeutic options for cryptosporidiosis are limited and only one drug, nitazoxanide, has been approved by the Food and Drug Administration (FDA). Efficacy is variable, with resolution of diarrheal symptoms and parasitological cure in 50–90% of HIV-negative children and adults (Rossignol et al., 1998, 2001; Amadi et al., 2002; Hussien et al., 2013). Typical oral doses are 500-mg twice daily for adults and adolescents, 200-mg doses twice daily for children aged 4–11 years, and 100-mg doses twice daily for children aged 1–3 years, with treatment duration of 3–14 days. The drug is generally well tolerated. Notably, placebo treatment resulted in symptom improvements in a significant fraction (30–40%) of patients (Rossignol et al., 2001; Amadi et al., 2002), underlining that the infection is usually self-limited in immunocompetent patients. In contrast, nitazoxanide is not effective in HIV-infected children (Amadi et al., 2002), and is not FDA approved for this indication.

Other pharmacological interventions have been used clinically against *Cryptosporidium*, but they are not FDA-approved for treating cryptosporidiosis and their efficacy is generally lower than nitazoxanide. For example, paromomycin (500 mg oral, four times daily for 2–3 weeks) led to symptom cessation and microbiological cure in 60–70% of immunocompetent patients (Vandenberg et al., 2012; Hussien et al., 2013). Results in HIV-infected adults have been variable, with some studies showing similar (60–70%) efficacy of paromomycin (Bissuel et al., 1994; Blanshard et al., 1997), while others reporting no effectiveness (Hewitt et al., 2000). Immunotherapy has shown some promise. Most notably, restoration of immune competence in immunocompromised hosts, particularly an increase toward normal in the CD4 T cell levels in patients with AIDS, is associated with clearance of *Cryptosporidium* (Flanigan et al., 1992). Passive immunotherapy by oral administration of colostrum-derived bovine immunoglobulins (which presumably contained anti-cryptosporidial antibodies as cows get commonly infected with *Cryptosporidium*) led to significant improvement of diarrheal symptoms, although microbiological cure was not evaluated (Greenberg and Cello, 1996). However, nitazoxanide remains the most effective current therapeutic agent available against cryptosporidiosis in immunocompetent individuals, while no consistently effective drugs exist against infection under conditions of immunodeficiency.

### Assays for Drug Development

Antimicrobial drug development depends on suitable assays that recapitulate critical growth phases of the microbe in the host. This is not usually a problem for growth autonomous microorganisms, such as most bacterial pathogens, but can be a challenge for symbionts such as *Cryptosporidium* that rely partially or completely on host cells for growth and survival. Nonetheless, an array of cell culture and animal models is available for drug testing against the parasite.

**FIGURE 2:**
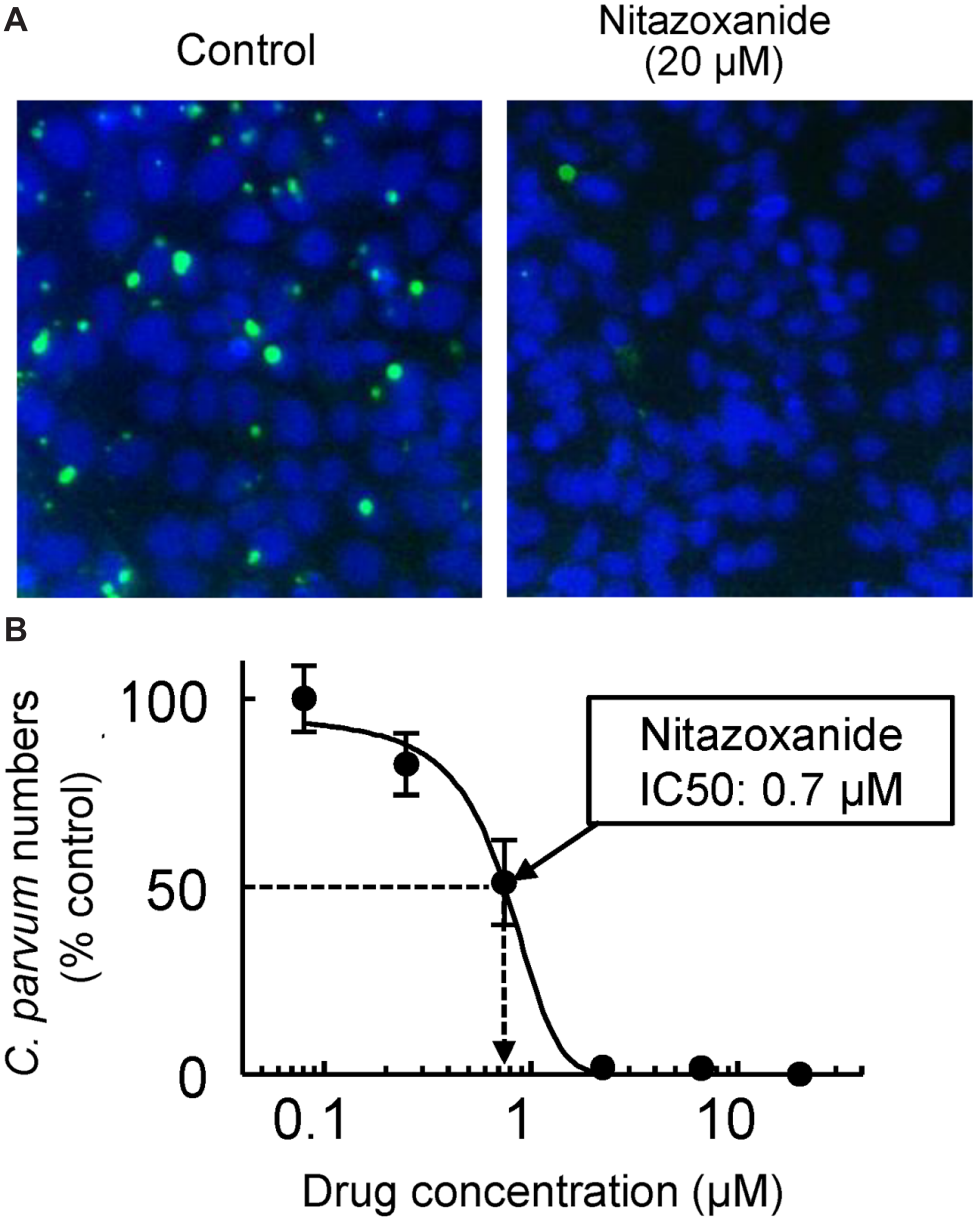
***In vitro* drug assay for *Cryptosporidium*.** HCT-8 human intestinal epithelial cells were grown as monolayers in 96-well plates to 60–80% confluence. *Cryptosporidium parvum* oocysts, pretreated for 10 min at 37°C with 10 mM HCl and 10 min at 15°C with 200 μM sodium taurocholate to promote excystation, were added to the wells (10^5^ oocysts/well). After a 4 h infection period, monolayers were washed, serial dilutions of nitazoxanide were added, and cultures were incubated for 48 h at 37°C in 5% CO_2_ and 95% air. Cells were fixed with 3% formaldehyde, permeabilized with Triton X-100, and stained with FITC-conjugated *Vicia villosa* lectin to detect *C. parvum* and counterstained with Hoechst 33342 to detect nuclei. A representative image of host cell nuclei (blue) and parasites (green) is shown in **(A)** for controls (left) and after exposure to 20 μM nitazoxanide (right). A concentration-response curve is shown in **(B)**. Data are mean ± SD of triplicate wells, and are expressed relative to the parasite load in untreated controls. The half-maximal inhibitory concentration (IC50) for nitazoxanide was 0.7 μM.

Asexual and sexual development of *Cryptosporidium* can be achieved *in vitro* using several different cell lines, although the life cycle is incomplete and the number of oocysts produced is generally low. A widely used model is the human ileocecal adenocarcinoma cell line, HCT-8 (Upton et al., 1994; Alcantara Warren et al., 2008). Monolayers of the cells can be readily infected in culture (**Figure 2**), and infection can be optimized by adding nutritional supplements to the culture media (Upton et al., 1995). Other mammalian cell lines (e.g., MDCK and Vero) are also infectable, although parasite development tends to be less robust than in HCT-8 cells (Upton et al., 1994). Several of the epithelial cell lines, including human T84 and Caco-2 cells, can be grown as differentiated polarized monolayers on microporous filter supports, which permits studies of the effects of *Cryptosporidium* infection on barrier functions, ion absorption and secretion, and cytokine responses (Griffiths et al., 1994; Laurent et al., 1997, 1998). A recent report has also used primary human intestinal epithelial cells, grown as self-sustaining organoids (enteroids), for *in vitro* infections with *Cryptosporidium* (Castellanos-Gonzalez et al., 2013a). These cultures, unlike epithelial cell lines, display the normal range of intestinal epithelial cell differentiation, which should allow more complete analysis of parasite–host interactions than possible in transformed cell lines, although establishment and maintenance of primary cultures are technically demanding at this time and may not yet be readily adaptable to high-throughput screens. Overall, epithelial cell cultures are poised to remain an indispensable tool for new drug development against *Cryptosporidium*. It must be borne in mind, however, that current culture systems probably do not capture all stages of the cryptosporidial life cycle with equal efficiency and physiological impact, so drug screening efforts may be skewed toward compounds that are particularly active in the intracellular life cycle stages, but not any extracellular stages (Hijjawi et al., 2002).

Antimicrobial activity *in vitro* is usually necessary for a new drug candidate, but it is not sufficient since many candidates that work *in vitro* fail to be active *in vivo*. Appropriate animal models are therefore critical for drug development. A number of mammals have been used for *Cryptosporidium* infections, including small (mice, rats) and large animals (pigs, cows), each with advantages and disadvantages. Most animal models reproduce the cryptosporidial growth cycle and lead to sustained infection and shedding of infectious oocysts, which is adequate for drug efficacy studies. Small animal models, particularly mice, are resource-effective, but are not associated with the characteristic infection-induced diarrhea seen in humans and can thus not be used for investigations of this clinical manifestation and its treatment. On the other hand, large animals such as piglets and calves develop marked diarrhea (Tzipori et al., 1981, 1983; Argenzio et al., 1993; Gookin et al., 2008; Adell et al., 2013; Operario et al., 2015), but require greater resources and are less amenable to genetic and pharmacological manipulations for mechanistic studies.

Mice are the most commonly used models for *in vivo* testing of drug candidates against *Cryptosporidium*. Newborn mice are susceptible to infection with *C. parvum* during the initial 3 weeks after birth, but subsequently become resistant to infection. Consequently, adult wild-type mice are generally not suitable for drug testing, which is presumably due to effective innate or acquired immune defenses. However, adult mice with selected immunodeficiencies can be readily infected with the parasite. For example, SCID mice (Kuhls et al., 1992) and mice deficient in IFN-γ or its receptor (Theodos et al., 1997; von Oettingen et al., 2008), major histocompatibility complex class II (Aguirre et al., 1994), CD40 or CD40 ligand (Cosyns et al., 1998; Hayward et al., 2001), or IL-12 p40 (Urban et al., 1996; Campbell et al., 2002) are all susceptible to intestinal *C. parvum* infection and have been used to varying degrees for drug testing. The observation that nitazoxanide has efficacy in immunocompetent patients, but less so or not at all in immunodeficient individuals suggests that intact host immunity contributes to drug effectiveness. It follows that the nature of an immune defect that renders adult mice susceptible to *Cryptosporidium* may impact the predictive value of *in vivo* drug testing. However, few if any comparative drug efficacy studies have been done between different murine models, so it is not clear whether any particular model is more representative of the human situation than others and thus potentially more predictive of clinical efficacy.

### New Agents against *Cryptosporidium*

Antimicrobial drug development commonly proceeds along one of two pathways, focused either on whole-cell activity or on specific molecular targets. In an activity-centered approach, compound libraries are screened for activity against the live parasite in a suitable *in vitro* assay. Upon identification of biologically active “hits”, the respective compounds are systematically optimized for *in vitro* and ultimately *in vivo* activity. In a target-centered approach, the focus is on exploiting molecular targets that are indispensable and, ideally, unique to the parasite. Target identification may come from genomic analyses or genetic screens, and is followed by pharmacological exploration of their “druggability” with suitable inhibitors. Both approaches have advantages and shortcomings. Activity-centered drug development can rapidly lead to strong hits and lead compounds, but if those fail in subsequent preclinical or clinical trials, development might run into a dead end because the molecular target is often not known can thus not be further exploited with alternative chemical scaffolds. Target-centered drug development is conceptually compelling, as it promises systematic exploitation of genome information without the uncertainty of large library screens. However, targets may not prove as physiologically critical as initially predicted, and development of specific inhibitors with adequate activity *in vitro* and *in vivo* may not be straightforward. Furthermore, tools for genetic manipulation of *Cryptosporidium* are not presently available, precluding genetic screens as a strategy for identifying critical target genes in this parasite. To date, activity-centered approaches have yielded most if not all clinically utilized antimicrobials, while target-centered approaches remain an intriguing but unfilled promise.

Both drug development strategies have been applied to *Cryptosporidium*. In particular, the obligate endosymbiotic growth phase has sparked great interest in the systematic exploitation of this apparent “Achilles’ heel” of the parasite. *Cryptosporidium* has degenerate mitosomes instead of mitochondria and has lost the mitochondrial genome and nuclear genes for many mitochondrial proteins, including those required for the tricarboxylic acid cycle, oxidative phosphorylation, and fatty acid oxidation (Abrahamsen et al., 2004; Xu et al., 2004). Also lacking are genes for *de novo* biosynthesis of amino acids, nucleotides, and sugars (Abrahamsen et al., 2004; Xu et al., 2004). Loss of genes from multiple metabolic pathways indicates that the parasite relies heavily on scavenging nutrients from the host, salvage rather than *de novo* biosynthesis, and glycolysis or substrate-level rather than oxidative phosphorylation for energy production (Ryan and Hijjawi, 2015). Consequently, the parasite must depend on these essential core metabolic pathways, which are absent in or highly divergent from those in humans and animals, making many of the enzymes in these pathways potential drug targets.

Perhaps the most extensively studied metabolic pathway for drug development is the salvage of purines for nucleic acid synthesis. *Cryptosporidium* cannot synthesize purine nucleotides *de novo*, but instead converts adenosine salvaged from the host into guanine nucleotides via a pathway involving inosine 5′-monophosphate dehydrogenase (IMPDH; Umejiego et al., 2004). This enzyme catalyzes the conversion of inosine-5′-monophosphate into xanthosine-5′-monophosphate as the rate-determining step in guanine nucleotide biosynthesis. Interestingly, the parasites appear to have obtained their IMPDH gene by lateral gene transfer from bacteria, making it structurally distinct from mammalian IMPDH enzymes (Striepen et al., 2004). The exclusive reliance on this guanine salvage pathway makes IMPDH a potential drug target. Indeed, generic inhibitors of the enzyme have significant anticryptosporidial activity *in vitro* (Striepen et al., 2004). Subsequent studies have systematically advanced the development of more specific inhibitors. Based on high throughout screens, a number of structurally diverse IMPDH inhibitors were identified (Umejiego et al., 2008), and several of the most promising scaffolds were then explored for optimal activity *in vitro* and *in vivo*. For example, a number of derivatives of urea, benzoxazole, and benzopyrano[4,3-*c*]pyrazole are active against purified IMPDH in the low to mid nM range (Gorla et al., 2012, 2013; Sun et al., 2014), and several have activity against *C. parvum* in cell culture and in a mouse infection model *in vivo* (Gorla et al., 2014). As an alternative strategy to conventional hit identification and lead optimization of small “drug-like” molecules, peptides have been explored as IMPDH inhibitors. In particular, phylomer peptides, which represent naturally stable protein segments from phylogenetically diverse bacterial genomes with an evolutionarily optimized ability to bind protein surfaces (Watt, 2009), have been screened for inhibitory activity against *C. parvum* IMPDH (Jefferies et al., 2015). Peptides that interact with IMPDH were identified in a yeast two hybrid screen, and then tested for parasite inhibition *in vitro*. The best peptides suppressed *C. parvum* growth with 50% efficiency at 8–46 μM, but had only negligible cytotoxicity in human cells (Jefferies et al., 2015). Although the *in vivo* efficacy of these peptides remains to be established, the data underline that IMPDH is a valid pharmacological target for parasite inhibition. Taken together, the recent studies suggest that further preclinical development of IMPDH inhibitors, using either current scaffolds or perhaps yet to be discovered ones, holds great promise for a new class of anticryptosporidial agents.

Several other molecular targets have been explored against *Cryptosporidium*. For example, the *C. parvum* genome encodes three long chain fatty acyl-coenzyme A synthetases, which are essential in the fatty acid metabolism of the parasite (Guo et al., 2014). Their inhibition with triacsin C was highly effective against parasite growth *in vitro* and reduced oocyst production by 88% in infected IL-12 knockout mice (Guo et al., 2014). Another target, calcium-dependent protein kinase 1 (CDPK1), plays a role in calcium-mediated signaling of the parasite, which is important for regulating vital functions in *Cryptosporidium* and other apicomplexan parasites (Murphy et al., 2010; Ojo et al., 2010). Inhibition of CDPK1 with pyrazolopyrimidine derivatives blocked enzyme activity *in vitro* in the low nM range and parasite growth in epithelial cells in the low μM range (Murphy et al., 2010). Moreover, at least one of the derivatives exhibited significant efficacy after oral dosing in a mouse infection model (Castellanos-Gonzalez et al., 2013b). A third group of molecular targets are cysteine proteases, which have been associated with excystation and host cell invasion of *C. parvum* (Nesterenko et al., 1995; Perez-Cordon et al., 2011). Selective protease inhibitors have shown promising activity against the parasite *in vitro* and *in vivo* (Kang et al., 2012; Ndao et al., 2013), although it remains to be established which out of the twenty or more members of the papain-like family of cysteine proteases are responsible for these therapeutic effects.

Beyond target-centered drug development, several activity-centered drug screening approaches have been undertaken for *Cryptosporidium*. For example, a library of 727 FDA-approved drugs or drug-like compounds with a history of use in human clinical trials was tested for activity on parasite growth in HCT-8 epithelial cells (Bessoff et al., 2013). Approximately twenty compounds, representing a hit rate of ∼3%, showed significant activity at <10 μM, with the best compounds displaying *in vitro* IC50s in the range of 0.2–5 μM (Bessoff et al., 2013). Mechanistic pursuit of one of these leads revealed that the drug target is 3-hydroxy-3-methyl-glutaryl-coenzyme A (HMG-CoA) reductase, which is required for the *de novo* synthesis of isoprenoids (Bessoff et al., 2013). Importantly, the parasite lacks all known enzymes required for the synthesis of isoprenoid precursors, so inhibition of the critical enzyme in the host blocks parasite growth because *Cryptosporidium* is dependent on the host cell for synthesis of isoprenoid precursors (Bessoff et al., 2013). This finding nicely supports the concept mentioned above that the obligate endosymbiosis of the parasite is a potential “Achilles’ heel” that may be exploited for drug development. It must be noted, however, that drugs that exclusively target host processes also have an increased risk for adverse effects, so it may be more difficult for such drugs compared to conventional microbe-targeted drugs to find an acceptable balance between parasite clearance and other drug effects.

In another screen, an existing library from the Medicines for Malaria Venture Open Access Malaria Box was tested for activity against *C. parvum* (Bessoff et al., 2014). The library represents a collection of 400 compounds selected from 19,000 structurally unique molecules with activity against the erythrocytic stage of *Plasmodium falciparum* in three large phenotypic high-throughput screening campaigns undertaken by large pharmaceutical companies. The screen yielded 19 compounds, representing a ∼5% hit rate, with significant activity against *C. parvum* growth in HCT-8 cells at 6.6 μM (Bessoff et al., 2014), suggesting that the relatively high hit rate of the *Plasmodium*-active compounds against *Cryptosporidium* may be attributed to the general similarities between the two apicomplexan parasites. The most active compounds belonged to three chemical series, derived from the quinolin-8-ol, allopurinol-based, and 2,4-diaminoquinazoline, and several of the derivatives exhibited submicromolar potency *in vitro* (Bessoff et al., 2014). Further studies will have to explore their *in vivo* efficacy.

### Outlook

Taken together, recent studies clearly demonstrate that new classes of antimicrobials against *Cryptosporidium* are biologically possible and pharmacologically feasible, either by advancing leads against already identified targets or by screening new and larger compound libraries for activity against the parasite. In addition, analyses of the genomes and metabolomes of different *Cryptosporidium* species and genotypes are likely to reveal more molecular drug targets. However, as scientifically promising as drug development against *Cryptosporidium* is at this time, the major challenge for these efforts is the underlying economics (Striepen, 2013). As a disease that affects mostly developing countries and which had up to recently been significantly underestimated in its clinical prevalence and importance (Lal and Hales, 2015), cryptosporidiosis remains in the category of Neglected Diseases with low funding priority and limited commercial interest. If a new urgency in medical progress against the disease can be established at national funding agencies or philanthropic organizations, meaningful and timely progress could be made in treating and possibly preventing cryptosporidiosis.

## Giardia

Giardiasis is a major cause of diarrheal illness worldwide with hundreds of millions annual cases. Compared to cryptosporidiosis, giardiasis is often associated with more prolonged, chronic diarrhea and disease even in immunocompetent individuals, which, particularly in children, can lead to long-term sequelae including intellectual deficits and failure to thrive. Furthermore, treatment options are currently broader than those for cryptosporidiosis, but resistance to existing antimicrobial drugs is becoming a clinical problem that has not yet been described for cryptosporidiosis.

### Parasite

**FIGURE 3:**
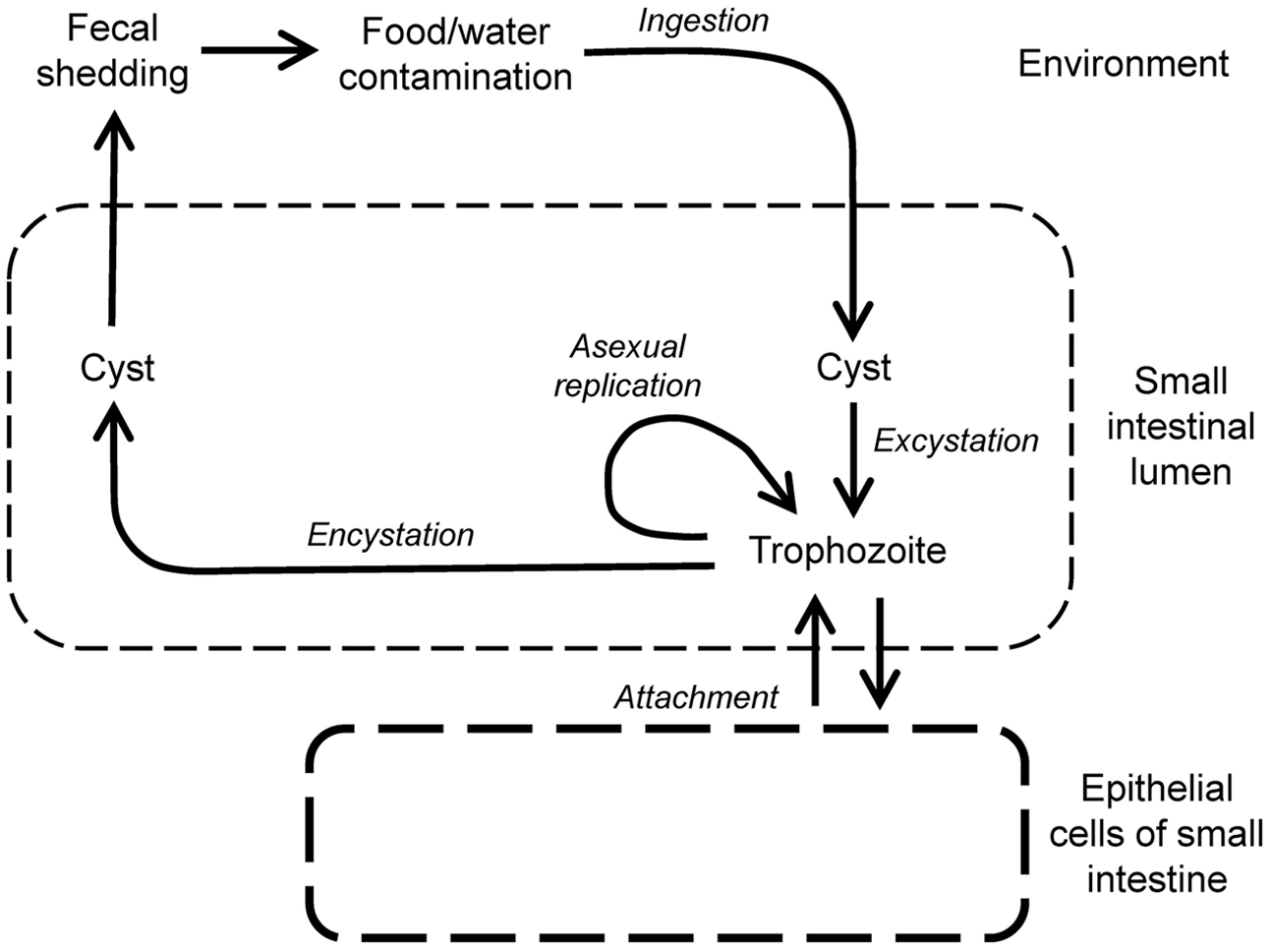
**Life cycle of *Giardia lamblia*.** Infection begins by oral uptake of cysts, which excyst in stomach and small intestine to form trophozoites, the replicating, disease-causing form of the parasite. Trophozoites are motile, and maintain their presence in the small intestine by regulated attachment to and detachment from the small intestinal epithelium, but they do not invade epithelial cells. Trophozoites can also differentiate by encystation in the small intestine into cysts, which are shed in the feces and are infectious for new hosts.

Giardiasis is caused by *Giardia lamblia* (syn. *G. duodenalis* or *G. intestinalis*), which was first discovered in the 1600s by van Leeuwenhoek in his own stool. *G. lamblia* is a flagellated, enteric parasite that belongs to the order of diplomonads in the phylum of metamonads, a large group of flagellate amitochondriate protozoa. The parasite has a comparatively simple life cycle (**Figure 3**), which occurs in a single host (monoxenous) and involves only two forms, the infectious cyst and the replicating trophozoite. Cysts are spread through contaminated drinking water or food, or person-to-person contact. *G. lamblia* is highly contagious, as evidenced by experimental human infection after oral ingestion of as few as 10 cysts (Nash et al., 1987). The low infectious dose, combined with the resistance of cysts to many common disinfectants (Betancourt and Rose, 2004; Erickson and Ortega, 2006), makes *G. lamblia* a constant threat to the safety of public water supplies. After passing through the stomach, trophozoites emerge from the cysts and colonize the upper small intestine. The sequence of exposure to an acidic environment, followed by neutral conditions and the presence of bile is sufficient to trigger excystation, as has been shown *in vitro* (Boucher and Gillin, 1990).

The haploid genome of *G. lamblia* comprises ∼12 Mb of DNA encoding ∼5,000 genes (Morrison et al., 2007; Franzen et al., 2009). The parasite is polyploid throughout its life cycle. Trophozoites have two nuclei, each carrying a diploid genome, so the overall genome is tetraploid (4N) in the trophozoite stage (Bernander et al., 2001). During encystation, the genome content acquires 16N ploidy through two successive rounds of chromosome replication without intervening cell division. Upon excystation, the cells have four nuclei, each with a ploidy of 4N, and divide twice to form four trophozoites containing two diploid nuclei each (Bernander et al., 2001). Besides the polyploidy, evidence exists for allelic sequence heterozygosity within single parasites (Ankarklev et al., 2012), further complicating the interpretation of genome information, as well as the use of genetic tools for identification of potential new drug targets. Moreover, *G. lamblia* displays considerable genetic diversity. Isolates are grouped into genetic Assemblages, A–F, with all human pathogens belonging to groups A and B, while the others are pathogens in dogs, birds, and other species. Genome comparison of isolates from Assemblage A and B shows only 77% nucleotide and 78% amino acid identity in protein-coding regions (Franzen et al., 2009), indicating that these two assemblages truly represent different *Giardia* species, despite the historic convention of a single species name (Jerlstrom-Hultqvist et al., 2010). Importantly, Assemblage A and B strains infect humans throughout the world, making it critical that new drugs are active against divergent *G. lamblia* strains of both Assemblages.

### Pathogenesis and Disease

*Giardia lamblia* causes hundreds of millions of annual cases of diarrheal disease in endemic and epidemic fashion worldwide (Karanis et al., 2007; Baldursson and Karanis, 2011). In the United States, the parasite is one of the two most common causes of outbreaks of parasitic disease, with prevalence rates of 1–7% depending on the population sampled (Furness et al., 2000; Barry et al., 2013). Infections are more frequent and severe in young children, particularly in day-care centers, and among travelers, hikers, and military personnel in the field (Overturf, 1994; Sharp et al., 1995). Following cyst ingestion, symptoms occur after a 1–2 weeks incubation period, although only about half of all stool-positive *Giardia* infections are symptomatic. Frequent clinical manifestations of giardiasis include watery diarrhea, epigastric pain, nausea, and vomiting. The acute phase typically lasts 1–3 weeks, although some patients can have persistent symptoms for months. Most infections are self-limited but recurrences are common in endemic areas. Chronic infections can lead to weight loss and malabsorption and may be mistaken for inflammatory bowel disease or anorexia nervosa (Thomas Iv et al., 2014). Importantly, chronic giardiasis is associated with stunting (low height for age), wasting (low weight for height) and cognitive impairment in children in developing countries (Berkman et al., 2002; Nematian et al., 2008; Al-Mekhlafi et al., 2013), a finding that has been reproduced in murine models of infection (Bartelt et al., 2013). Furthermore, acute giardiasis may disable patients for extended periods and can elicit protracted post-infectious syndromes, including irritable bowel syndrome and chronic fatigue (Hanevik et al., 2014).

After emergence from cysts, the flagellated *G. lamblia* trophozoites colonize the upper small intestine, although colonization of the lower small intestine, as well as stomach and colon can occur. Trophozoites reside and replicate in the intestinal lumen and at the intestinal epithelial surface, but do not invade deeper layers of the mucosa. The parasite’s ability to attach to the mucosal surface is critical for its persistence in the host (Lauwaet et al., 2010), yet the underlying mechanisms remain poorly defined. The ventral disk of the parasite can assume a domed conformation that is important for robust attachment (Woessner and Dawson, 2012), whereas flagellar motility is important for positioning and orienting trophozoites prior to attachment, but is not directly required for attachment (House et al., 2011). Despite its “off-shore” location outside the mucosa, the parasite actively engages mucosal immunity, although frank inflammation is typically absent in most cases (Oberhuber et al., 1997). However, villus and brush border microvillus atrophy are common, leading to digestive enzyme deficiencies (Solaymani-Mohammadi and Singer, 2011), and chronic giardiasis can lead to overt mucosal inflammation with pronounced villus loss (Hanevik et al., 2007).

Giardiasis is self-limiting in >85% of cases in non-endemic areas, indicating that effective immune defenses exist. Furthermore, symptoms of giardiasis are much less severe in endemic than non-endemic regions, suggesting gradual build-up of immunity (Faubert, 2000). Secretory IgA (Langford et al., 2002; Davids et al., 2006), intestinal hypermotility (Andersen et al., 2006; Li et al., 2006), and antimicrobial peptides and nitric oxide (Eckmann et al., 2000; Tako et al., 2013) have all been shown to have direct effector activity against the parasite, although their relative contributions to clearance of *Giardia* may be variable (Singer and Nash, 2000a; Langford et al., 2002). Beyond direct effectors, several immune cells and regulators are known to be involved in antigiardial immune defense. Mast cells and CD4^+^ T cells, but not CD8^+^ T cells, are required for clearing *Giardia* infection (Singer and Nash, 2000a; Roxstrom-Lindquist et al., 2006; Solaymani-Mohammadi and Singer, 2010). CD4^+^ T cells may act in part by controlling antigiardial IgA responses (Heyworth, 1989), while their functions are not related to classical Th1 or Th2 subsets, since their signature cytokines, IFN-γ, or IL-4, play no role in immune defense (Singer and Nash, 2000a). In contrast, IL-6 and IL-17 are important in *Giardia* clearance (Bienz et al., 2003; Zhou et al., 2003; Dreesen et al., 2014; Dann et al., 2015). IL-6 appears to act by promoting dendritic cell functions during infection (Kamda et al., 2012), although it has many other activities, including activation of neutrophils and monocytes, enhancement of follicular helper T cell responses, and stimulation of B cell proliferation and antibody production.

A human vaccine against giardiasis is not available. A crude veterinary vaccine (GiardiaVax), composed of total cell lysates of a mixture of sheep, dog and human isolates, reduces symptoms and duration of cyst output in cats and dogs (Olson et al., 2000). Interestingly, the vaccine has also been used as an immunotherapeutic agent in dogs with chronic giardiasis that had failed standard drug treatment (Olson et al., 2001), raising the intriguing possibility that a vaccine may be effective post-exposure.

### Current Treatment Options

Several classes of antimicrobial drugs are available for the treatment of giardiasis. The most commonly utilized worldwide are members of the 5-nitroimidazole (5-NI) family such as metronidazole and tinidazole. However, this first line therapy fails in up to 20% of cases and cross-resistance between different agents can occur (Gardner and Hill, 2001), and resistance to all major antigiardial drugs has been reported (Ansell et al., 2015). Alternative agents exist for treatment failures and for special circumstances (e.g., pregnancy), but these are generally less effective than 5-NI drugs (Escobedo and Cimerman, 2007).

The oldest and most common 5-NI, metronidazole, is typically given in three divided daily 250 mg oral doses for 5–10 days, and has a reported efficacy of 80–95% (Gardner and Hill, 2001). More recently, tinidazole, first approved by the FDA in 2004, has become a good alternative for giardiasis because of its efficacy (85–90%), tolerability, and convenience (a single oral dose is recommended; Speelman, 1985). All 5-NI drugs are prodrugs whose microbial specificity is due to the requirement for reduction to toxic free radical intermediates by low redox potential reactions present only in the anaerobic target microbes (Upcroft and Upcroft, 2001). The metabolism of *G. lamblia* is fermentative, and electron transport proceeds in the absence of mitochondrial oxidative phosphorylation. However, the parasite is microaerotolerant and can reduce molecular oxygen and thus protect the highly oxygen-sensitive, central metabolic enzyme, pyruvate:ferredoxin oxidoreductase (PFOR), and iron-containing ferredoxins. PFOR decarboxylates pyruvate and donates electrons to ferredoxin, which in turn reduces other components in the electron transport chain and leads to ATP generation. Reduced ferredoxin can also reduce the critical nitro group of 5-NI prodrugs to toxic radicals which kill the parasite. Other reduction pathways, including nitroreductases and thioredoxin reductase, also activate 5-NI drugs in *Giardia* (Pal et al., 2009; Leitsch et al., 2011; Nillius et al., 2011), although their relative importance in reducing metronidazole and other nitro drugs remains to be established. The radicals that result from nitro drug reduction form covalently bonded adducts on microbial target molecules, leading to their inactivation. The specific molecular targets of 5-NI drugs have not been defined in *G. lamblia*. In spite of the general efficacy of 5-NI drugs, treatment failures in giardiasis are common (up to 20%), clinical resistance is proven, and *in vitro* resistance can be induced so that parasites grow in clinically relevant levels of metronidazole (Wright et al., 2003; Tejman-Yarden et al., 2011).

Nitazoxanide is a nitrothiazole with broad-spectrum activity against intestinal parasites. Like 5-NI drugs, it is a prodrug that must be reduced to form toxic radicals, which inactivate various microbial target molecules including PFOR (Hoffman et al., 2007). It is usually given in two daily 500 mg doses for 3 days, which is more convenient dosing than metronidazole, but has slightly lower efficacy (70–80%) than 5-NI drugs (Gardner and Hill, 2001) and is also impacted by metronidazole resistance (Tejman-Yarden et al., 2011). In a clinical trial involving children with diarrheal illness, nitazoxanide reduced symptom duration in those aﬄicted with giardiasis as well as in those without a microbiological diagnosis (Rossignol et al., 2012), further underlining its utility as a broad-spectrum antimicrobial agent.

Benzimidazoles, such as albendazole and mebendazole, are generally used to treat intestinal helminth infections. Albendazole is also effective in giardiasis (Gardner and Hill, 2001; Solaymani-Mohammadi et al., 2010), although its efficacy varies markedly (25–90%) depending on the dosing regimen. Albendazole can be taken once daily for 5 days, making it more convenient than three-times-a-day metronidazole, and its anti-helminth activity makes it an attractive agent for dual use purposes (Granados et al., 2012). However, results from a Bolivian cohort study (a region with endemic *Giardia* and helminth infections) found that treatment with mebendazole reduced hookworm infections but paradoxically led to an increase in giardiasis (Blackwell et al., 2013), suggesting an antagonistic relationship between the two parasites and complicating the prospect of multi-target therapies.

Quinacrine, an old malaria drug, is an acridine derivative with excellent efficacy against giardiasis (90%). A recent report describes the experience of a family of four with giardiasis, three of whom failed therapy with tinidazole but were cured with quinacrine (Requena-Mendez et al., 2014). In a randomized trial in children with giardiasis, chloroquine was equally effective as tinidazole and superior to albendazole (Escobedo et al., 2003). Despite its good efficacy, quinacrine has potentially severe adverse effects, including a number of psychiatric and dermatologic manifestations, and is no longer commercially available in the United States or Canada.

Finally, paromomycin is an aminoglycoside with antigiardial activity *in vitro* (Edlind, 1989) and *in vivo* (Geurden et al., 2006). However, it less effective among adults with metronidazole-refractory disease than other therapies and is rarely used in clinical practice.

Therapeutic strategies for treatment-refractory giardiasis include longer duration and/or higher doses of the original agent, switching to a different class of drug, or combination therapy. In a case series of 10 patients who failed nitroimidazole therapy, all were cured with one of the following combinations: metronidazole or tinidazole + paromomycin + albendazole in three cases, metronidazole + paromomycin in two cases, tinidazole + paromomycin in two cases, tinidazole + quinacrine in two cases, and metronidazole + quinacrine in one case (Morch et al., 2008). All the drugs were administered for 7 or 10 days except for tinidazole, which was given for 1–7 days. The combinations were well tolerated and had no serious adverse effects. In a case report, a HIV patient with giardiasis was unsuccessfully treated five times with metronidazole and albendazole, but was cured with nitazoxanide 500 mg b.i.d for 10 days and then 1 g b.i.d. for 15 days (Abboud et al., 2001). Susceptibility testing showed the strain to be resistant to metronidazole and albendazole, but susceptible to nitazoxanide (Abboud et al., 2001). Combination therapy generally decreases the risk of developing antimicrobial drug resistance, although it is presently not known how this concept applies to giardiasis.

### Assays for Drug Development

**FIGURE 4:**
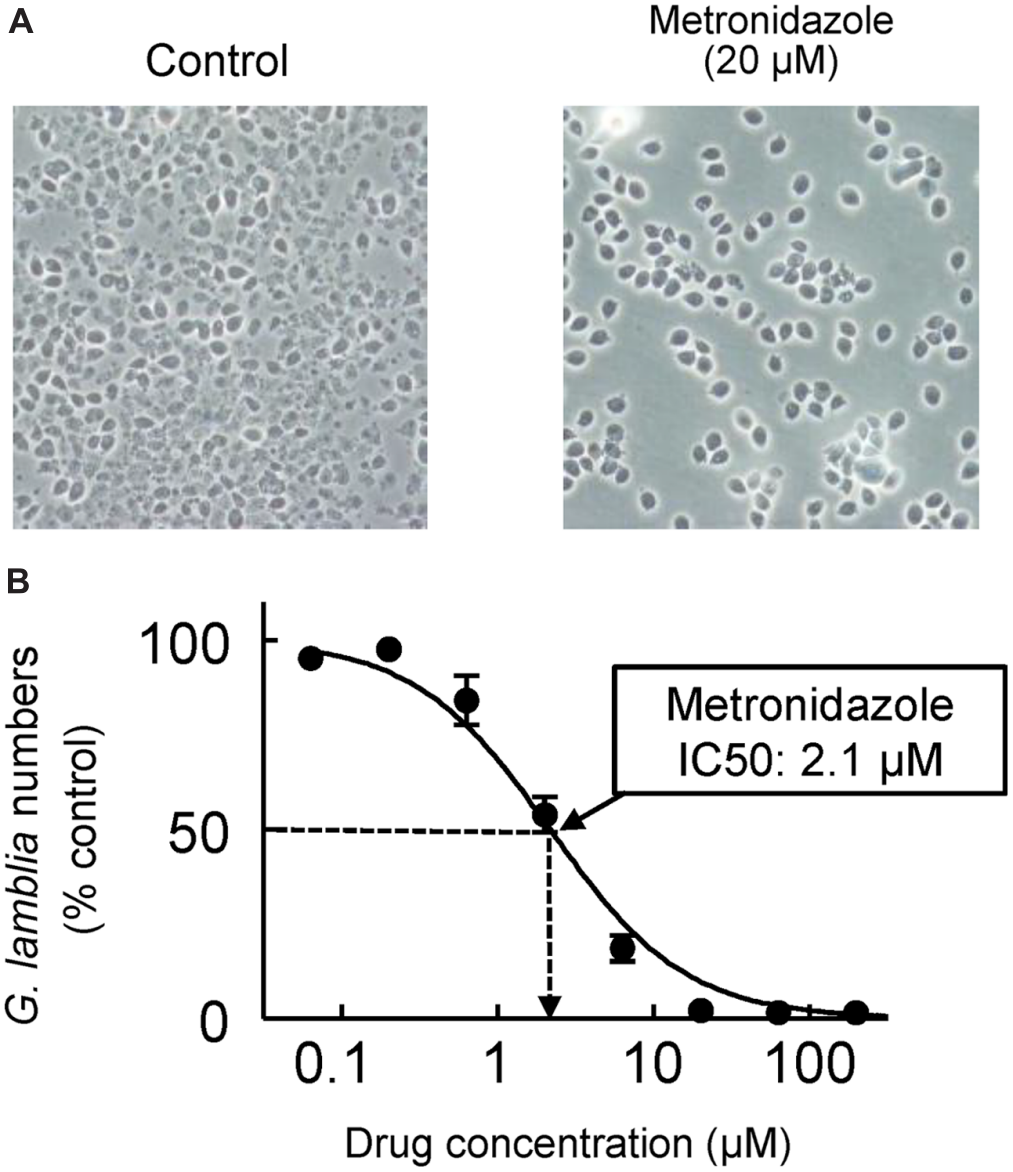
***In vitro* drug assay for *G. lamblia*.** Serial dilutions of test compounds were made in 96-well plates, and trophozoites of *G. lamblia* WB were added to the wells (2 × 10^3^/well). After 48 h incubation at 37°C under anaerobic conditions, cell growth and viability was determined with an ATP assay. **(A)** Representative phase-contrast images for controls without added drug (left) and cells exposed to 20 μM metronidazole (right). **(B)** A concentration-response curve for metronidazole. Data are mean ± SD of triplicate wells, and are expressed relative to the *G. lamblia* numbers in untreated controls. The IC50 for metronidazole was 2.1 μM in this experiment.

*Giardia lamblia* trophozoites can be readily grown *in vitro* under axenic conditions, which greatly simplifies drug testing compared to the endosymbiont *Cryptosporidium*. Typical assays for *Giardia* involve serial drug dilutions in multi-well plates and parasite growth for 48 h under anaerobic conditions (Valdez et al., 2009; Miyamoto et al., 2013) (**Figure 4**). Cell numbers and viability can be assessed microscopically (Gut et al., 2011), or by determining ATP content in the cultures (Valdez et al., 2009) (**Figure 2**). A number of Assemblage A and B isolates are available, and resistant lines against all major antigiardial drugs have been generated in the laboratory (Upcroft et al., 1996a,b; Dunn et al., 2010; Tejman-Yarden et al., 2011). To date, drug-resistant axenic lines have not been generated from clinical isolates derived from patients who failed therapy, although short-term animal studies of such isolates have shown that resistance occurs *in vivo* (Lemee et al., 2000).

Appropriate animal infection models are a key requirement for antimicrobial drug development. A number of mammals, including different rodents, dogs and cats, can be experimentally infected with *G. lamblia*. In cats and dogs, giardiasis has veterinary relevance (Bouzid et al., 2015), so drug development has veterinary objectives in these species. For drug testing studies against human *G. lamblia*, the most common models are mice and Mongolian gerbils. Adult mice are not readily infectable with most human *G. lamblia* strains, with the sole exception of the Assemblage B strain, GS/M (Byrd et al., 1994). The strain was shown to cause diarrheal disease upon experimental inoculation in humans (Nash et al., 1987), so it is a clinically relevant, pathogenic strain, yet the reasons for its seemingly unique ability to colonize adult mice remain unclear. However, following the observation that resistance to infection could be conferred by co-housing of animals, it was shown that conditioning of mice with a cocktail of suitable antibiotics (typically vancomycin + gentamicin or neomycin + ampicillin or penicillin; none of these kill *G. lamblia*) in the drinking water sensitizes mice to infection with a range of different *G. lamblia* strains of both A and B assemblages (Singer and Nash, 2000b; Solaymani-Mohammadi and Singer, 2011). This conditioning regimen is now commonly used, although the impact on drug testing has not been explored. As an alternative, gerbils can be infected with a wide range of human *G. lamblia* strains without antibiotic conditioning (Tejman-Yarden et al., 2011, 2013; Benere et al., 2012). They have proven valuable for pathophysiologic and immunological studies, but have not been widely used for drug testing.

### New Drugs

The discovery of antibiotics revolutionized medicine and represents a turning point in human history. Unfortunately, enthusiasm for these miracle drugs was soon tempered by the emergence of resistant strains. As in bacteria, protozoa like *Giardia* can develop resistance that make widely used agents ineffective. While still uncommon, antibiotic resistance (especially against 5-NI drugs) is increasing in *G. lamblia* and contributes to treatment failures (Lemee et al., 2000). Elucidating the complex resistance mechanisms is an active area of investigation and important insights have been gained over the last decade. Recent studies indicate that drug resistance in *G. lamblia* is caused by one of several different cellular adaptations. Of these, the best described mechanism is the loss of the parasite’s ability to activate nitro prodrugs to toxic radicals by reduction, thus effectively allowing the parasite to avoid suicidal drug activation. For example, suppression of PFOR leads to metronidazole resistance (Dan et al., 2000) and certain strains of metronidazole-resistant *G. lamblia* have reduced levels of this critical enzyme required for ferredoxin-dependent drug activation (Leitsch et al., 2011). Other drug activation pathways have been identified, and they can also be impacted in resistant cells (Pal et al., 2009; Leitsch et al., 2011; Nillius et al., 2011). Another mechanism of drug resistance to both metronidazole and nitazoxanide is altered expression of genes involved in stress responses, including heat-shock proteins and expression of nitazoxanide-binding proteins (Muller et al., 2008). Fortunately, metronidazole-resistant giardiasis is not yet common in clinical practice, perhaps because of the detrimental effects of metronidazole resistance on attachment and infectivity in a subset of organisms, although others with resistance can infect normally (Tejman-Yarden et al., 2011). Resistance to other antigiardial drugs also exists. For example, resistance to benzimidazoles is thought to occur through mutations in the molecular target, β-tubulin (Aguayo-Ortiz et al., 2013). Overall, the studies on emerging drug resistance and the underlying mechanisms underline that the development of new antigiardial agents to broaden the therapeutic armamentarium remains an important challenge.

The goals of new drug development against *G. lamblia* and *Cryptosporidium* are somewhat different. For *Cryptosporidium*, only one moderately effective drug (nitazoxanide) is available, so new classes of more effective drugs are a high priority. By comparison, for *G. lamblia*, several classes of drugs with good efficacy exist, but dosing regimens are suboptimal or emerging resistance begins to threaten clinical utility. Consequently, improvements in potency and dosing, and the ability to overcome existing and prevent new forms of drug resistance are priorities in antigiardial drug development. A number of strategies have been employed for the development of new drugs against *G. lamblia*, which include, as for *Cryptosporidium*, both activity- and target-centered approaches. In addition, optimization within existing drug classes, analogous to the development of next-generation β-lactam antibiotics, has played a much more important role in drug development against *G. lamblia* compared to *Cryptosporidium*.

**FIGURE 5:**
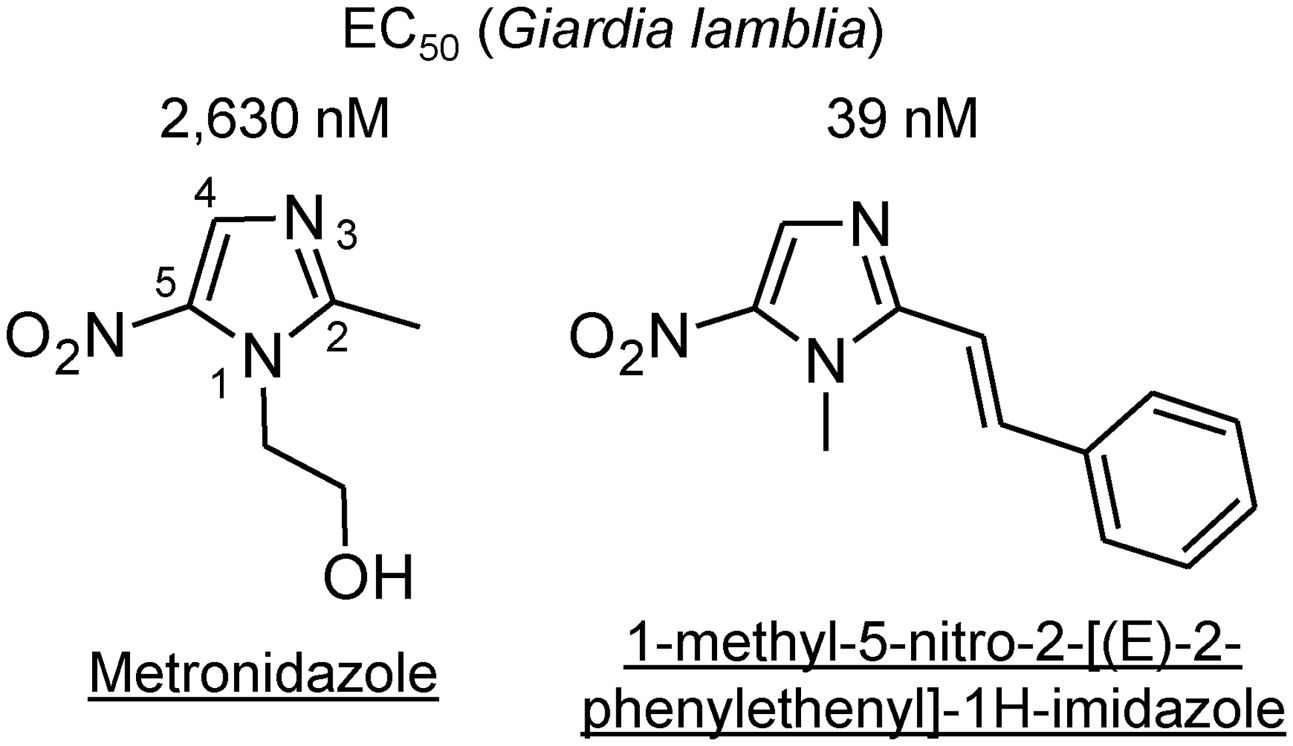
**Structures of 5-nitro antimicrobial drugs.** The structures of metronidazole (left) and a 2-position derivative are depicted, along with their respective *in vitro* EC50 values against a representative strain of *G. lamblia* (Valdez et al., 2009). The numbers along the core imidazole of metronidazole designate the ring positions.

Prime examples for the potential of optimization within an existing drug class are the 5-NI compounds. The most common 5-NI, metronidazole, is composed of a 5-membered nitroimidazole core carrying simple hydroxyethyl and methyl side chains in the 1′ and 2′ position, respectively (**Figure 5**). Modifications of these side chains have been a productive strategy for developing new and improved agents. For example, substitution of side chains in the 1′, 2′, or 4′ positions of the imidazole ring with larger and more complex functional groups has led to compounds with enhanced (up to 500-fold) antigiardial activity (Upcroft et al., 2006; Valdez et al., 2009; Miyamoto et al., 2013) (**Figure 5**). Importantly, many of the new 5-NI compounds were able to overcome existing forms of metronidazole resistance (Miyamoto et al., 2013). The underlying mechanisms for improved activity remain to be determined but may relate to differential drug activation by one or more reductases (Leitsch et al., 2011; Nillius et al., 2011). For other metronidazole derivatives it was shown that they were more active than the parent compound in altering the morphology and ultrastructure of *G. lamblia* by affecting vesicle trafficking and differentiation into cysts (Busatti et al., 2013). Similar advances have been demonstrated for the 5-nitrothiazole, nitazoxanide, where placement of a benzene ring between the nitro group and the thiazole ring was used to create new benzologues (Navarrete-Vazquez et al., 2011). The best compounds were five-times more active than nitazoxanide and 18-times more active than metronidazole against *G. lamblia* and showed minimal toxicity in mammalian cells (Navarrete-Vazquez et al., 2011). In another series of nitrothiazole derivatives, substitutions at 2′ position with a range of acylamino side chains yielded compounds with >10-fold greater activity against *G. lamblia* than the reference compound nitazoxanide (Nava-Zuazo et al., 2014). Collectively, these studies demonstrate that systematic structural modifications of existing nitro drugs are likely to yield vastly improved antimicrobials that can serve as “next-generation” nitro drugs for the treatment of giardiasis and potentially other infections with clinically important anaerobic pathogens (Miyamoto et al., 2013).

Similar modification strategies have been applied to benzimidazoles, whose prototype compound, albendazole, has been used as an alternative to 5-NI drugs against giardiasis. A number of novel derivatives have been created, including an alkylthiol group at the 2′ position and an ethoxy group at the 5′ position of the benzimidazole ring, resulting in highly potent compounds with antigiardial activity in the 10–50 nM range (Perez-Villanueva et al., 2013). Using reverse-phase high performance liquid chromatography and permeability assays with cultured epithelial cells, it was observed that the antigiardial activity of a series of benzimidazole derivatives was influenced by their lipophilicity, hydrogen bond donors, and molecular volume, but not by their apparent permeability across epithelial cell monolayers (Hernandez-Covarrubias et al., 2012). A computational analysis of the structure-activity relationships of multiple benzimidazoles showed complex activity landscapes for antigiardial activity, with substitution at position 2 on the benzimidazole moiety playing an important role in increasing potency and substitutions at positions 4–7 influencing selectivity against *G. lamblia* over other protozoan parasites (Perez-Villanueva et al., 2010). These findings offer hope that quantitative models may help to identify structural properties associated with enhanced antigiardial activity in future drug design, at least for compounds that have well-defined molecular targets such as β-tubulin for benzimidazoles (Aguayo-Ortiz et al., 2013). Similar structure activity analyses have been conducted for 5-NI derivatives (Miyamoto et al., 2013), although the current lack of understanding of the specific reductases involved in prodrug activation or the molecular targets of activated drugs confounds these analyses and complicates their exploitation in drug development.

Besides compound optimization within existing drug classes, activity-centered screens of drug libraries have also been applied to *G. lamblia*. For example, a screen of 1,520 compounds from the BIOMOL ICCB Known Bioactives 2 and NINDS Custom Collection 2 libraries for growth-inhibitory activity against *G. lamblia* yielded 48 initial hits, some of which, such as indirubin, were not known to kill *G. lamblia*, although many others had known antigiardial agents or were broad inhibitors of cellular functions (e.g., actinomycin D; Bonilla-Santiago et al., 2008). Such toxic compounds are not likely to be of clinical utility. By comparison, screening of libraries of FDA-approved drugs has the distinct advantage that preclinical safety testing has already been done, which can accelerate the progression to clinical trials of efficacy. For instance, screening of a library of 746 compounds available for human use found that auranofin, which is employed in the treatment of rheumatoid arthritis, inhibited the growth and survival of multiple different *G. lamblia* isolates (Tejman-Yarden et al., 2013). Importantly, auranofin completely overcame metronidazole resistance (Tejman-Yarden et al., 2013). The action mechanism involved inhibition of thioredoxin oxidoreductase, a critical enzyme involved in maintaining normal protein function and combating oxidative damage in *G. lamblia* and other parasites including *Entamoeba histolytica* and *Schistosoma mansoni* (Kuntz et al., 2007; Debnath et al., 2012; Tejman-Yarden et al., 2013). Given that its pharmacokinetic properties and toxicology are well established (Blocka et al., 1986), auranofin may be a promising new candidate for the treatment of drug-resistant giardiasis. In another screen, a total of 2,816 compounds from the Library of Pharmaceutical Active Compounds (LOPAC1280) and the NIH Chemical Genomics Center Pharmaceutical collection library (NPC) were tested for activity against *G. lamblia* (Chen et al., 2011). Detailed follow-up studies of 28 of the most active compounds identified three new drugs, fumagillin, carbadox, and tioxidazole, with strong activity against metronidazole-sensitive and -resistant *G. lamblia* isolates (Kulakova et al., 2014). Furthermore, fumagillin, a complex biomolecule derived from *Aspergillus fumigatus* with known antimicrobial activities against *Entamoeba* and microsporidia, was efficacious in a mouse giardiasis model (Kulakova et al., 2014).

An abbreviated form of activity-centered drug discovery is the retesting of compounds that have shown activity in related microbes. Screening of an NIH Chemical Genomics Center Pharmaceutical Collection containing >2,000 compounds identified 32 highly active compounds against *Plasmodium falciparum* (Yuan et al., 2011). One of these, tetrahydrolipstatin (orlistat), a lipase inhibitor approved for the treatment of obesity, was then tested for growth inhibition of *G. lamblia*, based on the general rationale that efficient lipid metabolism is important for a fast growing parasite such as *Giardia*. Orlistat showed a potent and dose-dependent inhibition of *G. lamblia* replication, with a half maximal inhibitory concentration significantly lower than that of metronidazole (Hahn et al., 2013). Notably, orlistat is poorly absorbed from the intestinal tract and remains highly active in the lumen until excreted in the stool, raising the possibility of essentially topical therapy of giardiasis. Disadvantages of orlistat are the potential malabsorption of lipids and other lipophilic drugs, as well as cost issues. Another example is miltefosine, a potent oral treatment for human visceral leishmaniasis due to *Leishmania donovani*. The drug had previously been shown to kill this parasite by inducing a cell death process with numerous cytoplasmic, nuclear, and membrane features of metazoan apoptosis (Paris et al., 2004). Subsequent testing of miltefosine against *G. lamblia* revealed good activity *in vitro* and efficacy in a mouse model *in vivo* (Eissa and Amer, 2012). As with orlistat, trials to ascertain the effectiveness of miltefosine in humans with giardiasis will be needed. More broadly, repurposing of existing human drugs can be a powerful and resource-efficient strategy for rapid progress in drug development, but the finite number of existing compounds (with only ∼1,500 currently FDA-approved drugs) ultimately limits this discovery path in the ongoing struggle for new antimicrobial agents.

One way out of the closed loop of drug repurposing is the screening of unknown chemical entities. Plants and their extracts have historically been rich sources for new antimicrobial agents. One of these is the naturally occurring weed *Oxalis corniculata*, long used in India for treating dysentery and diarrhea. Systematic fractionation of extracts was used to isolate a novel galacto-glycerolipid compound with antigiardial activity comparable to metronidazole (Manna et al., 2010). The compound had no toxicity in human cells (Manna et al., 2010). Extracts of sandalwood, *Osyris alba*, have been used by traditional healers in Jordan to ameliorate diarrheal diseases. Phytochemical investigation identified a new pyrrolizidine alkaloid, osyrisine, which had significant activity against *Giardia* but was non-toxic to human cells (Al-Jaber et al., 2010). These examples demonstrate that crude plant extracts, or perhaps in the future other rich biological sources of structurally diverse compounds, can be the source of new antigiardial compounds, although their medicinal characterization, synthesis, and optimization are likely to pose challenges not encountered with the exploration of libraries of chemically defined compounds with drug-like properties.

The third drug development strategy, exploitation of molecularly defined targets, has been less prominent for *Giardia* compared to *Cryptosporidium*. Nonetheless, a small number of *G. lamblia* targets have been explored for druggability. The metabolic enzyme arginine deiminase (ADI) catalyzes the first step in a major pathway for generating ATP in the parasite (Knodler et al., 1998). Knock-down of ADI by RNA interference resulted in non-viable trophozoites, indicating that ADI is important for trophozoite survival (Li et al., 2009). Furthermore, ADI may also serve as a virulence factor by depleting arginine in the host, which may enable *Giardia* to evade the host immune response (Eckmann et al., 2000; Banik et al., 2013). Humans lack an analogous enzyme to ADI, making it an attractive target for drug development. Another enzyme target is carbamate kinase, which catalyzes the final step in the arginine dihydrolase pathway converting ADP and carbamoyl phosphate to ATP and carbamate. It is essential in *Giardia* metabolism, but has no equivalent in humans (Minotto et al., 1999). A luminescent enzyme activity assay was employed in a high throughput screen of over 4,000 compounds to identify 30 hits with activity in the low mM range (Chen et al., 2012). Although the detected activities are low at this point, the hits could be starting points for the development of more potent and selective inhibitors of carbamate kinase in *G. lamblia* and ultimately a novel class of antigiardial drugs. A third attractive target enzyme in *Giardia* is fructose 1,6-bisphosphate aldolase (FBPA), which catalyzes the reversible cleavage of D-fructose-1,6-bisphosphate to dihydroxyacetone phosphate and glyceraldehyde 3-phosphate, a key step in the Embden–Meyerhof–Parnas glycolytic pathway used by amitochondriate protozoa as a main source of energy (Li et al., 2011). FBPA is critical for *Giardia* survival and had unique structural and functional features that distinguish it from human analogs (Galkin et al., 2007). Inhibitors of the enzyme have antigiardial activity in the 100 nM range and very low mammalian cytotoxicity (Navarrete-Vazquez et al., 2015).

### Outlook

Several treatment options currently exist for giardiasis, but important therapeutic challenges remain. One major objective is improvements in dosing, ideally in form of a single, highly effective oral dose, which is the ultimate goal for most if not all tropical infection diseases as it allows diagnosis and definitive treatment on a single clinic visit. Another objective is the ability to overcome and prevent drug resistance, which is not yet common for *G. lamblia*, but constitutes a continuous threat that should not be taken lightly. Current studies suggest that these objectives can be achieved within existing classes of drugs or with new classes of drugs and molecular targets. However, as for cryptosporidiosis, a major challenge are the economic incentives for new drug development against giardiasis, which is clinically important throughout the world but is typically considered a Neglected Disease with primary health impact in low-development countries (Escobedo et al., 2010).

## Conflict of Interest Statement

The authors declare that the research was conducted in the absence of any commercial or financial relationships that could be construed as a potential conflict of interest.
